# Examination of Combined Treatment of Ginsenoside Rg3 and 5-Fluorouracil in Lung Adenocarcinoma Cells

**DOI:** 10.1155/2022/2813142

**Published:** 2022-06-28

**Authors:** Kuizhong Shan, Yujiang Deng, Zhiquan Du, Peiyu Yue, Sufang Yang

**Affiliations:** Department of Pulmonary Nodule Center, The Second People's Hospital of Kunshan, Suzhou City 215300, China

## Abstract

Chemotherapy is a commonly used strategy for advanced lung cancer patients. However, its clinical application is restrained due to its toxicity and drug resistance. Ginsenoside Rg3 (Rg3) has a strong anticancer influence on colon cancer, breast cancer, lung cancer, and other malignant tumors. However, it is still unclear whether Rg3 can cooperate with 5-FU to inhibit the tumor growth and angiogenesis of lung adenocarcinoma (LUAD). This study examined the combined treatment of Rg3 and 5-FU in LUAD. It was revealed that the combined treatment could notably enhance the suppression on proliferative, invasive, and migratory abilities and angiogenesis in LUAD cells A549 and SPC-A-1. On the other hand, we also discovered that Rg3 or 5-FU could suppress the activity of the NF-*κ*B signaling pathway and downregulate VEGFA expression in LUAD cells. Collectively, this study suggested that Rg3 combined chemotherapy may perform a more powerful drug efficiency in LUAD cells.

## 1. Introduction

Chemotherapy or radiotherapy plus chemotherapy is commonly applied to the advanced LUAD patients [1, 2], where 5-fluorouracil (5-FU) is one of the chemotherapies for lung cancer [3]. Though 5-FU has achieved great progress in cancer treatment, its clinical application is restricted due to the development of drug resistance after chemotherapy [4, 5]. Previous studies showed that 5-FU combining other drugs can reduce its required dose, ensuring the effect of chemotherapy while reducing the adverse reactions caused by 5-FU [4, 6]. Hence, it is of consequence to further study the pathogenesis of LUAD and to find a more valid and less toxic drug to be combined with 5-FU to strengthen the chemotherapeutic efficacy of 5-FU. A key factor in the current trend toward personalized medicine is the correct understanding of causes and mechanisms that lead to low or lack of sensitivity of tumor tissues to 5-FU-based therapies [7].

Various studies proved that Chinese medicine has distinctive advantages in the treatment of diseases, which is specifically manifested as more components, more targets, less adverse reactions, etc. [8]. Ginsenoside Rg3 (Rg3) is a positive monomer extracted from ginseng [9, 10], which is capable of promoting immune response and antitumor activity [11]. Rg3 is the most potent extract among steroidal saponins [12] and was proved to inhibit the growth of varying human cancers. For example, Wang et al. [13] found that 20 (s)-Rg3 facilitates apoptosis of ovarian cancer HO-8910 cells through XIAP and PI3K/Akt pathways. Yang et al. [14] demonstrated that Rg3 displays strong antitumor activity in colorectal tumors, and its effect is achieved by repressing C/EBP*β*/NF-*κ*B signal transduction. Rg3 suppresses the growth of colorectal tumor by downregulating the C/EBP*β*/nuclear factor- (NF-) *κ*B signaling pathway. Rg3 also inhibits endothelial cell proliferation and survival, and the expression of various factors involved in angiogenesis [15]. For instance, Sun et al. [16] showed that Rg3 notably restrains migration and proliferation of endothelial cells and reduces the expression of vascular endothelial development factor- (VEGF-) A protein, thereby inhibiting the progression of glioma. Clinically, Rg3 is often utilized to inhibit tumor angiogenesis. For example, Rg3 inhibits angiogenesis in endometriosis through the VEGFR-2-mediated PI3K/Akt/mTOR signaling pathway [17]. Meng et al. [18] demonstrated that Rg3 restrains proliferation and migration of endothelial cells by downregulating VEGF, so as to further inhibit melanoma angiogenesis. Hu et al. [19] found that Rg3 restrains activation of microtumor angiogenesis *in vivo* besides apoptosis inducing, suggesting that Rg3 can be employed as an adjunct to the clinical treatment of hepatocellular carcinoma (HCC). Based on the previous research, we assume that Rg3 is a promising drug worthy of further study to provide an effective therapeutic regimen for LUAD.

In this current study, the impacts of Rg3 and 5-FU on the anticancer activity and antiangiogenesis against LUAD cells were evaluated by *in vitro* cell functional assays. The potential molecular mechanism was also examined. This study may offer a new theoretical basis for LUAD treatment.

## 2. Materials and Methods

### 2.1. Cell Cultivation and Reagents

Human LUAD cell lines A549 (BNCC341254) and SPC-A-1 (BNCC100939) and the endothelial cell line HUVEC (BNCC337616) used in this study were all provided by BeNa Culture Collection (China). Endothelial cells were cultivated in CM15-1 medium containing 10% fetal bovine serum (FBS) (Lonza, USA). A549 and SPC-A-1 cells were cultivated with Roswell Park Memorial Institute- (RPMI-) 1640 medium (Sigma, USA) containing 10% FBS (HyClone, USA). All cells were cultivated in a 37°C incubator with 5% CO_2_. The endothelial cell line HUVECs (c-0230-5c) were purchased from Cascade Corporation (USA) and cultured in Medium 200 with a 2% low serum growth supplement (LSGS) (Thermo Fisher Scientific, USA).

Rg3 and 5-FU were acquired from Sigma (USA) and were formulated utilizing dimethyl sulfoxide (DMSO) (Sigma, USA). In cell experiments, Rg3 or 5-FU mother liquor was diluted with DMSO upon meeting the required concentration and then dripped into cells and cultured with cells for 48 h.

### 2.2. CCK-8 Assay

A549 and SPC-A-1 cells were placed into 24-well plates at a density of 5 × 10^5^ cells/well for incubation of 4 h. The cells were then cultivated with 10% CCK-8 regent for 2 h. Optical density (OD) was determined at 450 nm by a microplate reader (BioTek Instruments Inc., USA).

### 2.3. Colony Formation Assay

A549 and SPC-A-1 cells were put into 6-well plates at a density of 0.5 × 10^3^ cells/well for 10 days of culture. Subsequently, colonies were fixed with 10% formaldehyde for 5 min and stained with 1% crystal violet for 30 s. Finally, colonies were photographed and counted.

### 2.4. Transwell Assay

For migration measurement, cells were added to the transwell chambers at a density of 1 × 10^5^ per well. For invasion determination, the matrix glue was diluted with medium and 1 × 10^5^ cells per well were cultivated in transwell chambers. Invaded or migrated cells were immobilized and stained with crystal violet for 15 min. Finally, images of 4 visual fields were taken under a microscope (BX53, Olympus, Japan).

### 2.5. Angiogenesis Assay

A549 and SPC-A-1 cells were cultured in culture solution treated with different concentrations of Rg3 (0, 0.25, 0.75, and 1 mmol/L) and 5-FU (0, 25, 50, and 100 *μ*mol/L). Then, the supernatant was collected and conditioned medium (CM) was prepared. HUVECs were cultured in fibrin glue to form a capillary-like tubular structure model *in vitro.* CM was added to the glue, and the area of vascular formation and the number of vascular connections formed by endothelial cells were observed and recorded.

### 2.6. Nuclear and Cytoplasmic Extraction Assay

NE-PER Nuclear and Cytoplasmic Extraction Reagents (Thermo Fisher Scientific, USA) were utilized to separate cytoplasmic and nuclear components from LUAD cells. Next step, proteins in the cytoplasm and nucleus were extracted in line with the specifications and further analyzed by western blot.

### 2.7. Western Blot

A549 and SPC-A-1 cells were lysed in lysis buffer. Subsequently, the extracted proteins (10-30 *μ*g) were separated on 8-10% sodium dodecyl sulfate-polyacrylamide gel electrophoresis and transferred to a polyvinylidene fluoride membrane (Millipore, USA). The blocked membranes were cultivated with primary antibodies and HRP-conjugated secondary antibody. Observations were made utilizing the electrochemiluminescence (ECL) system (Thermo Fisher Scientific, USA). Specific information about the antibodies applied in our study is presented in Supplementary Table 1. GAPDH was utilized as the control for total proteins and cytoplasm, while PARP was the control for nuclear protein.

### 2.8. Data Analysis

The experimental data were processed by GraphPad Prism 6.0 (GraphPad Prism 6.0, USA). The outcomes were presented in the form of mean ± standard deviation. Student's *t*-test was employed for comparison between two groups. *P* < 0.05 was thought to be statistically significant. Each assay was repeated at least three times.

## 3. Results

### 3.1. Combined Treatment of Rg3 and 5-FU Performed More Effective Inhibitory Effects on Proliferation, Migration, and Invasion of LUAD Cells

**Figure 1:**
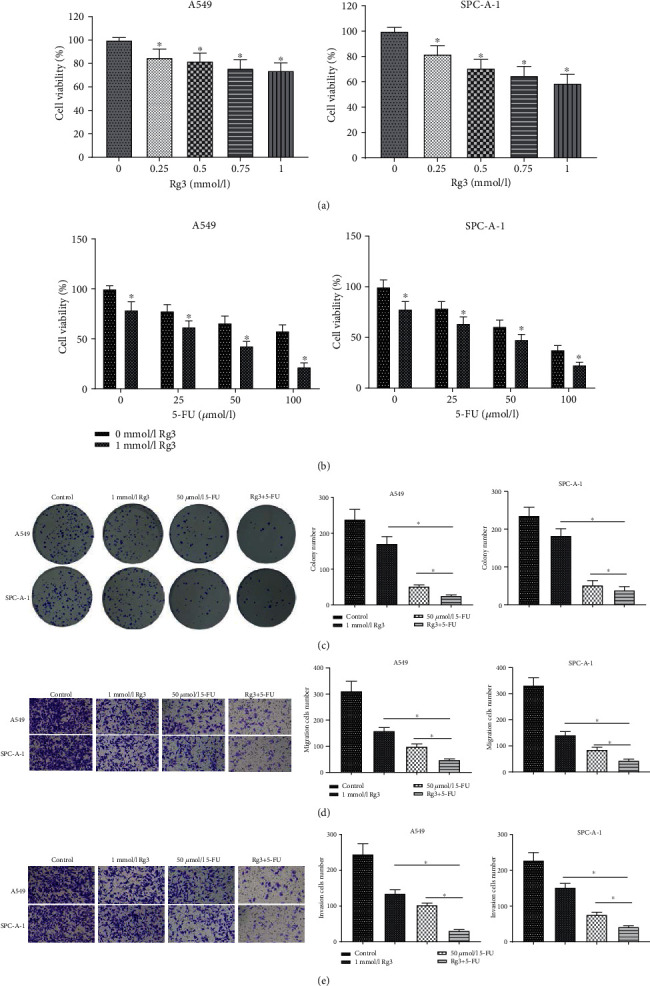
Combined processing with Rg3 and 5-FU facilitates suppression on the progression of LUAD cells. (a, b) CCK-8 assay measured the viability of A549 and SPC-A-1 cells processed by different concentrations of Rg3 or 5-FU; (c–e) after A549 and SPC-A-1 cells were treated with Rg3 (1 mmol/L), or 5-FU (50 *μ*mol/L) or both, the proliferation, migration, and invasion of A549 and SPC-A-1 cells were detected by colony formation assay (c) and transwell assay (d, e), respectively (∗ denotes *P* < 0.05).

After cells were processed with different concentrations of Rg3 or 5-FU, the CCK-8 method was employed to detect cell viabilities. As the result revealed, viabilities of A549 and SPC-A-1 cells were decreased remarkedly with the increase of Rg3 concentration (Figure 1(a)). The viabilities of the cells treated with Rg3 and 5-FU simultaneously were evidently declined in comparison with the viabilities of the cells treated by 5-FU alone. Moreover, viabilities of A549 and SPC-A-1 cells expressed a decreasing trend with the growth of 5-FU concentration as well (Figure 1(b)). However, the viabilities of SPC-A-1 cells treated by 1 mmol/L Rg3 and 100 *μ*mol/L 5-FU were lower than 25%, unfavorable to the subsequent western blot assay. Therefore, in the subsequent cell function assays, the concentrations of Rg3 and 5-FU were selected as 1 mmol/L and 50 *μ*mol/L, respectively. As the results of colony formation showed, the number of colonies formed in cells processed by Rg3 or 5-FU alone was prominently reduced in comparison with that of cells processed by Rg3 and 5-FU simultaneously (Figure 1(c)). Similarly, the transwell assay indicated that compared with Rg3 or 5-FU processed alone, the ability to migrate and invade migration and invasion of the cells treated by the two together was evidently declined (Figures 1(d) and 1(e)). These findings suggested that the combination treatment presented relatively more powerful inhibitory effects on proliferative, migratory, and invasive capacities of LUAD cells.

### 3.2. The Combined Treatment Activates the Inhibitory Effect on Angiogenesis of LUAD Cells

**Figure 2:**
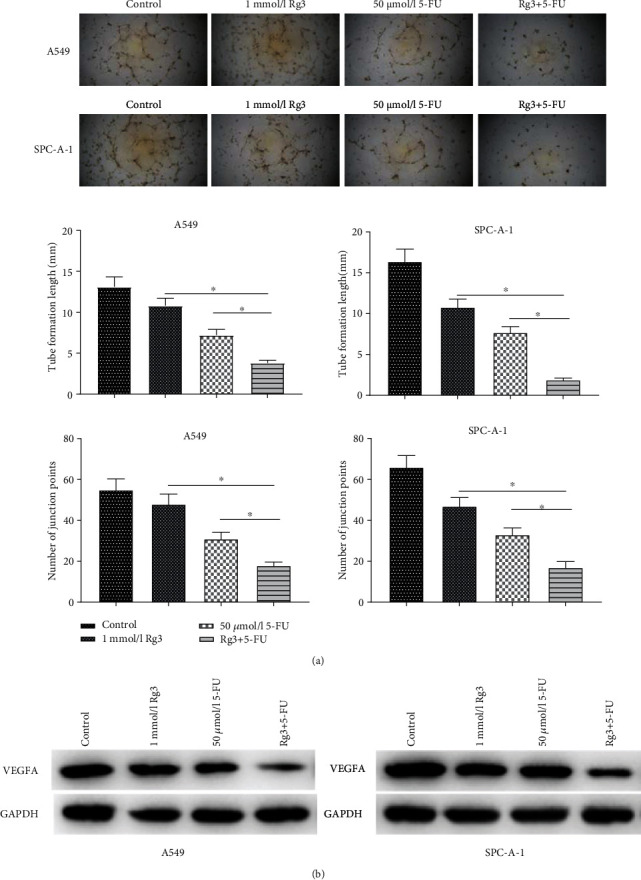
Combined processing with Rg3 and 5-FU boosts the inhibitory effect on angiogenesis of LUAD cells. After A549 and SPC-A-1 cells were treated with Rg3 (1 mmol/L), or 5-FU (50 *μ*mol/L) or a combination of them, (a) angiogenesis assay showed the angiogenic ability of the cells; (b) western blot expressed the protein expression level of VEGFA in LUAD cells A549 and SPC-A-1 (∗ denotes *P* < 0.05).

Angiogenesis of tumor cells was reported to be crucial to cell proliferation, migration, and invasion [20]. Therefore, to investigate the effects of Rg3 and 5-FU on angiogenesis of LUAD cells, HUVECs were treated with 1 mmol/L Rg3, 50 *μ*mol/L 5-FU, and 1 mmol/L Rg3+50 *μ*mol/L 5-FU, respectively. These cells were cultured in a LUAD cell-conditioned medium. Then, they were cultured in the matrix-coated pores for 2 days to establish capillary tubes. It was revealed that the vascular formation ability of HUVECs processed by RG3 and 5-FU was remarkably lower than that treated with RG3 or 5-FU alone, as evidenced by a random count of the vascular area of the cells and the number of vascular connections (Figure 2(a)). These results collectively indicated that Rg3 and 5-FU had a notably inhibitory effect on angiogenesis in HUVECs. It was reported that the VEGF family of endothelial growth factors is a crucial factor in the modulation of angiogenesis [21], and VEGFA plays a more dominant role in promoting angiogenesis and enhancing vascular permeability. Hence, we detected the protein expression of VEGFA in A549 and SPC-A-1 cells of each treatment group by western blot assay. The result indicated that the protein expression of VEGFA in LUAD cells was suppressed after treatment with Rg3 or 5-FU. In comparison with the Rg3 or 5-FU processing alone, VEGFA protein expression in the cells after the combined processing of the two was markedly reduced (Figure 2(b)). Together, the above experiments demonstrated that the combined treatment more effectively restrained VEGFA protein expression and inhibited tumor angiogenesis in LUAD.

### 3.3. The Combined Treatment Presents the Optimal Inhibitory Effects on VEGFA Expression and NF-*κ*B Signaling Pathway Activity in LUAD Cells

**Figure 3:**
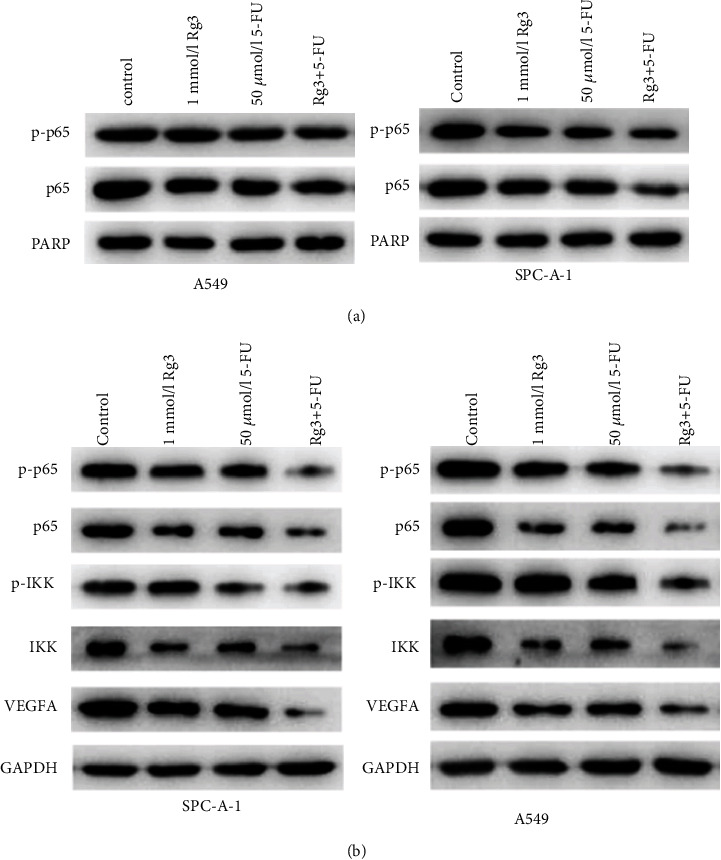
Combined processing with Rg3 and 5-FU inhibits VEGF expression and NF-*κ*B signaling pathway activity in LUAD cells. (a) Protein expression of nuclear protein p-p65 and p65 examined by western blot; (b) protein expression of p-p65, p65, p-IKK, IKK, and VEGFA in cells examined by western blot (∗ denotes *P* < 0.05).

*In vitro* cell functional experiments confirmed that combined processing with Rg3 and 5-FU could boost the suppressive effect on proliferation, migration, and invasion of LUAD cells. Angiogenesis assay uncovered that combined processing with Rg3 and 5-FU could restrain tumor angiogenesis by markedly reducing the expression of VEGFA. It was reported that the NF-*κ*B signaling pathway can induce the expression of related genes involved in tumor transformation and progression, thereby regulating tumor metastasis and angiogenesis [22, 23]. Hence, we further investigated whether combined treatment with Rg3 and 5-FU downregulated VEGFA expression by regulating the NF-*κ*B signaling pathway. Firstly, the expression of NF-*κ*B signaling pathway-related proteins was evaluated by western blot. The result revealed that in comparison with the control group, p-p65 and p65 expression levels in the nuclear protein extractions treated with Rg3 or 5-FU were decreased, while those in the nuclear protein extractions treated with Rg3 and 5-FU were markedly lower than those in the group processed by Rg3 or 5-FU alone (Figure 3(a)). Moreover, it was also found that p-p65, p65, p-IKK, IKK, and VEGFA expression levels in the total protein extractions of cells treated with Rg3 and 5-FU were the lowest (Figure 3(b)). The above-mentioned inhibitory effect could be hastened by combined processing with Rg3 and 5-FU. These results indicated that the combination therapy had the strongest inhibitory effect on VEGFA expression and NF-кB signaling pathway activity in LUAD cells.

## 4. Discussion

LUAD is one of the major histological types of lung cancer. Despite advances in conventional therapies (chemotherapy, molecular targeted therapy, and surgery), the 5-year survival rate of LUAD patients is less than 15% [24, 25]. 5-FU is a normally applied chemotherapy drug in clinical practice, and it is also an antimetabolite [26]. It has obvious prohibitive influences on proliferation, migration, and invasion of tumor cells. Nevertheless, at the same time, 5-FU has certain side effects. It is toxic to normal cells and can cause serious adverse reactions and even endanger the patients' survival, which severely restricts its clinical application [27, 28]. Therefore, it is of great significance to find a drug that can boost the chemotherapeutic efficacy of 5-FU and weaken the toxic and side effects of 5-FU for effective treatment of LUAD.

The effects of cancer therapies could be improved by combining them with traditional Chinese medicine (TCM). For different courses of cancer therapy, TCM could be used as adjunctive ingredients to polish cancer therapy [29]. Ginsenoside is the active ingredient of the traditional Chinese medicine ginseng, which plays an antitumor role in many cancers, including colon cancer [30]. However, its role in LUAD has not been reported, so we hypothesized that Rg3 could be used as an auxiliary component to enhance the therapeutic effect of 5-FU. After performing a series of experiments, we found that Rg3 could repress proliferation, migration, and invasion of LUAD cells. The combination of Rg3 and 5-FU showed a more effective inhibitory effect on the progression of LUAD cells.

Angiogenesis is the formation of new blood vessels from existing blood vessels, which is a pivotal process in the proliferation, migration, and differentiation of cancer cells. Since angiogenic factors control tumor growth and metastasis [31], angiogenic regulation of this process is critical for determining novel cancer treatment strategies [32]. VEGF plays a major part in the process of angiogenesis. VEGF increases vascular permeability by binding to its receptors, causes extravasation of plasma proteins and fibrins along with changes in the extracellular matrix, and migrates endothelial cells, laying a foundation for neovascularization [21]. Meng et al. [18] uncovered that Rg3 downregulates VEGF in melanoma cells to suppress proliferation and migration of endothelial cells, thereby restraining melanoma-induced angiogenesis. Our results suggested that combined processes by Rg3 and 5-FU notably inhibited VEGFA in LUAD cells. The combination of Rg3 and 5-FU activated the inhibition of angiogenesis in LUAD cells.

NF-*κ*B is an inactive dimer and binds to regulatory protein inhibitors of the *κ*B (I*κ*B) family. When NF-*κ*B is stimulated, I*κ*B is phosphorylated and then degraded by proteases. The released dimer of NF-*κ*B translocates to the nucleus and binds to the promoter or target gene, which binds to the enhancer *κ*B site, thereby activating NF-*κ*B and playing a transcriptional regulatory role [33]. It was reported that the NF-*κ*B signaling pathway plays a paramount part during the process of tumor cell metastasis, and its role in angiogenesis is achieved by regulating the inflammation response and VEGF expression [34–36]. Here, it was uncovered that p65 and p-p65 expression levels in the NF-*κ*B signaling pathway in LUAD cells were notably suppressed after combined processing with Rg3 and 5-FU. Expression levels of p-p65, p65, p-IKK, IKK, and VEGFA in LUAD cells processed by Rg3 and 5-FU were the lowest. These results together indicated that both Rg3 and 5-FU could constrain the activity of the NF-*κ*B signaling pathway and downregulate VEGFA. The above-mentioned inhibitory effect could be hastened by combined processing with Rg3 and 5-FU.

Viewed in total, this study investigated the functions of combined processing with Rg3 and 5-FU on the biological characteristics of A549 and SPC-A-1 cells through *in vitro* experiments. We uncovered that the combined processing with Rg3 and 5-FU not only fostered the inhibitory effect on proliferative, migratory, and invasive capacities of LUAD cells but also notably suppressed the angiogenesis of the cells. Besides, we also revealed that Rg3 combined with 5-FU could boost its inhibition on the NF-*κ*B signaling pathway and downregulate VEGFA in LUAD cells. Collectively, this investigation suggested that Rg3 boosted the anticancer effect of 5-FU on LUAD cells through the NF-*κ*B signaling pathway.

## Data Availability

The data used to support the findings of this study are included within the article.

## Supplementary Materials

Supplementary Materials Supplementary Table 1: information of antibodies utilized in this investigation.
