# Supplementary material for: Examination of Combined Treatment of Ginsenoside Rg3 and 5-Fluorouracil in Lung Adenocarcinoma Cells
Source: Comput Math Methods Med. 2022 Jun 28;2022:2813142. doi: 10.1155/2022/2813142 (PMC9256322; doi:10.1155/2022/2813142)
Supplement: Supplementary Materials — Supplementary Table 1: information of antibodies utilized in this investigation. [file 2813142.f1.doc]

**Supplementary Table 1 Information of antibodies utilized in this investigation**

| **Antibody** | **Specificity** | **Company** |
| --- | --- | --- |
| **p65** | Rabbit Polyclonal | Cell Signaling Technology, Beverly, MA, USA |
| **p-p65** | Rabbit Polyclonal | Cell Signaling Technology, Beverly, MA, USA |
| **VEGFA** | Rabbit Polyclonal | Abcam, Cambridge, London, England |
| **IKK** | Rabbit Polyclonal | Cell Signaling Technology, Beverly, MA, USA |
| **p-IKK** | Rabbit Polyclonal | Cell Signaling Technology, Beverly, MA, USA |
| **PARP** | Rabbit Polyclonal | Proteintech, Wuhan, Hubei, China |
| **GAPDH** | Rabbit Polyclonal | Proteintech, Wuhan, Hubei, China |
| **IgG H&L (HRP)** | Rabbit Polyclonal | Proteintech, Wuhan, Hubei, China |
